# Emergency engineering reconstruction mode based on the perspective of professional donations

**DOI:** 10.3389/fpsyg.2023.971552

**Published:** 2023-01-17

**Authors:** Hanyu Li, Xinli Zhang, Usama Khaliq, Faheem Ur Rehman

**Affiliations:** ^1^School of Management and Engineering, Nanjing University, Nanjing, Jiangsu, China; ^2^School of Business, Sichuan University, Chengdu, China; ^3^Graduate School of Economics and Management, Ural Federal University, Ekaterinburg, Russia

**Keywords:** cumulative excess return, double differential, multiple collaborative, emergency engineering, reconstruction mode

## Abstract

**Introduction:**

In the construction of emergency engineering, the effective participation of organizations such as government and construction enterprises can improve engineering emergency services to emergency adaptive, which faces the challenge of the enthusiasm of enterprises’ emergency participation. This study proposed a new diversified social governance mode for public emergency facility construction.

**Methods:**

This paper empirically analyzes the performance of stock market returns before and after construction enterprises’ participation in emergency engineering.

**Results:**

Against the backdrop of COVID-19, the analysis based on the sample data of 141 listed companies found that both construction enterprises directly involved in emergency hospital construction and non-state-owned enterprises involved in donations have higher excess return rates. In contrast, social concern has a positive mediating effect between emergency donations and stock market returns. The study suggests that stock market returns from donation behavior and reputation capital become incentives for firms to actively participate in emergency donations, providing a behavioral basis for professional donations by construction firms.

**Discussion:**

Based on the above assumptions, this paper proposes the organizational model of emergency engineering construction and the “Engineering Community” relationship based on the “Engineering Multi-governance” theory. This paper is the first to study the emergency engineering construction model from the perspective of professional donation behavior.

## 1. Introduction

Natural and man-made disasters frequently occur worldwide, and emergency engineering is an indispensable key factor in preventing and restoring the impact of disasters (Bai et al., 2006). In the construction of emergency engineering, the prominent feature is the tight construction time and heavy tasks. The conventional engineering management organization model faces significant challenges in completing the whole process of design, financing, and construction in a short time, with difficulties such as construction material mobilization and construction technology response (Supernaw, 2019; Sun and Xu, 2021; Wang et al., 2021). A new emergency engineering construction mode should be explored to promote the efficiency and sustainability of emergency services.

Wearne (2000) pointed out that if urgency means working faster than is justified by the financial criteria for sanctioning the project, all resources should be mobilized to shorten the duration. Once a disaster occurs, organizations and governments with the same experience should provide help and support for the first time. Wearne (2002) considered that workers with relevant construction experience should be sought to deal with any possible contingencies and changes during project construction. Previous researchers proposed promoting cooperation between government and enterprises, and government leadership and enterprise participation can effectively improve the construction efficiency of emergency projects (Pribadi et al., 2018; Kong and Sun, 2021). The above studies show that the government and construction enterprises are the main bodies constructing emergency projects. In terms of construction mode, scholars Fan (2022) proposed adopting the EPC model, in which the government assigned engineering tasks to the general contractor for design, procurement, construction, and other whole process management to improve efficiency and clarify responsibilities. Chen L.K. et al. (2021) showed that the construction of emergency projects could be accelerated by employing contract terms, such as penalties for exceeding deadlines and rewards for shortening deadlines. Nanjun et al. (2021), taking the rapid construction of Vulcan Mountain and Thunder Mountain hospitals in COVID-19 as an example, and the concept of an “anti-epidemic construction community” was proposed, that is, under the common goal, all participants in the project cooperate to complete the emergency project construction according to a close group composed of different layers and functions. In addition, construction technology is a crucial element in determining efficiency. Scholars Chen, Luo and Tan showed that the techniques serving for project success include methods such as building information modeling (BIM), modular composite building fabrication and assembly design (DfMA), and so on (Luo et al., 2020; Chen L.K. et al., 2021; Tan et al., 2021). Information technology has greatly improved construction efficiency. In general, emergency engineering is not an immediate deliverable but a process of building services, which requires a large amount of good investment in funds, materials, and technology. The existing literature mainly focuses on selecting construction contractors and the “government-enterprise” partnership but not on the supply mode of many engineering resources and services.

In the study of emergency management, there is a mature model for emergency material supply, namely the emergency donation model. Medical institutions and social organizations donated masks, protective clothing, and other materials during the COVID-19 (Bin-Nashwan et al., 2020). During the WenChuan earthquake, the community donated food and drinking water, and other necessities to the earthquake area (Huang et al., 2011). The material donation mode dramatically improves the speed of emergency response and relieves the short-term financial pressure on the government. In turn, enterprises can improve their reputation and competitiveness by fulfilling social responsibility, which has become a strategic donation for enterprises (Mahmud et al., 2021). Islam et al. (2013) pointed out that it is convenient for companies to make monetary donations but not for victims of disasters. It is more direct and effective to donate goods to the victims. In combination with their professional fields, professional donations carried out by enterprises have become a new research hotspot in emergency donations (Zhao et al., 2021).

Similarly, in the construction of emergency engineering, it is of great significance for construction enterprises with a professional background to make engineering donations instead of simple monetary donations or material donations. Let construction enterprises take the initiative to make engineering professional donations and carry out strategic donations that match the nature of the enterprise and the donation content, which can provide a basis for donation behavior incentives and emergency engineering construction. Taking the emergency engineering construction of Vulcan Mountain and Thunder Mountain as examples, this study examines the economic effects of different donation behaviors carried out by construction enterprises from the perspective of their financial benefits after response. Combined with the construction organization activities of emergency engineering management, this study proposes a new model of efficient emergency engineering donation construction through professional strategic donations of construction enterprises, expanding the scope and professionalism, which provides new ideas for the governance of emergency engineering construction.

Therefore, the contribution of this paper mainly has the following two points. First, there is no research on the content of emergency donations and the economic effect after donation. This study finds this gap and makes up for this research gap. This study takes Vulcan Mountain, and Thunder Mountain as examples from the perspective of construction enterprises introduces donation content and the interaction between donation content and enterprise nature, and evaluates its impact on the economic effect of enterprise donation. At the same time, social attention is a mediator variable. Secondly, this paper enriches the theory of emergency management. Currently, most of the research on emergency engineering focuses on the progress of construction methods, such as modular construction, BIM application, etc., and there is little research on the organizational model. This paper proposes a new emergency construction management mode by coupling emergency construction with an emergency donation and discusses the possibility of further strategic development as a new mode.

The study includes Part II, Research Design; Part III, Empirical Analysis; Part IV, Discussion and Recommendations; and Part V, Conclusion.

## 2. Literature review and theoretical hypothesis

### 2.1. Theoretical development

In the past development, a corporate philanthropic donation has become the mainstream trend of corporate social responsibility; and the research on the impact of corporate charitable donation on corporate performance has been very rich (Muller and Kraussl, 2011; Gao et al., 2012; Liang and Renneboog, 2017; Zhou et al., 2021) and continues to increase (Azmi et al., 2021). According to the enterprise stakeholder theory, in addition to shareholders, some stakeholders also play key roles in the survival and development of an enterprise, such as consumers, suppliers, and governments. Since the behavior of stakeholders significantly affects corporate performance, whether a company fulfills its social responsibilities will bring about changes in its reputational capital, which in turn affects the behavior of stakeholders, such as consumers’ purchasing behavior and investors’ investment behavior etc. Stakeholder pressure can motivate businesses to make charitable giving (Zhou et al., 2021). Charitable donations by enterprises will trigger media coverage (Peress, 2014), increasing the amount of information and the speed of dissemination, effectively reducing information costs, correcting information asymmetry, and raising social attention (Peress, 2014; Zhao et al., 2021). And because of the financial behavior theory, investors are not entirely rational people (Eyster et al., 2019); they are more inclined to buy stocks they are familiar with or trust with limited attention, which can improve the stock market returns of donated companies (Barber and Odean, 2008). There have been studies on corporate donations and stock market reactions in the context of the COVID-19 epidemic. It is found that enterprises participating in donations during the epidemic have better stock market performance, and non-state-owned enterprises are more motivated to donate (Chao and Yuwei, 2021). However, Chen H. et al. (2021) is believed that the novel coronavirus has brought great uncertainty, resulting in negative short-term response of investors to COVID-19-related donations, especially in the case of severe local transmission of the epidemic.

Emergency management initially belongs to the research category of crisis management. Western scholars first put forward the research concept of crisis management, which extended from the medical field to the political field and spread to the management field. The “4R” model of crisis management includes the entire emergency management process, including Reduction, Readiness, Response, and Recovery, from prevention before an event to an event. The preparation and response at the time of the incident and then the recovery at the end of the event correspondingly different management strategies are adopted in different periods. In the past few decades, natural disasters such as earthquakes, floods, and epidemics of various infectious diseases have occurred frequently in China and worldwide. Emergency management of disasters has become more critical in national strategies. More emphasis is placed on local and social organizations involved in emergency response (Ainuddin et al., 2013; Adhikari et al., 2016; Elkady et al., 2022), the first to participate in emergency response. At the same time, in disaster emergency management in recent years, the “government-enterprise” cooperation model has gradually increased, and the proportion of enterprises participating has increased yearly (Kong and Sun, 2021). After Hurricane Katrina in the United States, the role of local private enterprises in disaster relief has become increasingly prominent (Lu, 2014); since the 2008 Wenchuan earthquake in China, a large number of enterprises have actively participated in emergency rescue and post-disaster reconstruction (Shi et al., 2016); from the 1995 Hanshin Earthquake to the 2011 East Japan Earthquake, Japanese companies’ awareness of disaster participation has been continuously enhanced, and their ability to participate in disasters has been significantly improved (Gao et al., 2012; Li, 2016).

It can be seen from the above that the participation of enterprises in disaster emergency management as their fulfillment of social responsibility can improve the efficiency of emergency management while improving enterprise performance and achieving a win-win situation for society and enterprises.

### 2.2. Hypothesis development

It has been shown that the market incentives for corporate donation are influenced by the nature of the firm and the donation contents (Zhang et al., 2016; Hoi et al., 2019; Zhao et al., 2021). In emergency donations, by taking construction enterprises as the object, this paper studies whether different donation contents bring about other stock market performance; whether enterprises participating in engineering donations have higher abnormal returns. At the same time, this paper further explores the interaction between the nature of equity of construction firms involved in donations and donation contents; whether firms of different natures should make different donations. It also introduces social concern as a mediating variable. The following hypotheses are proposed in this paper.

1. The different donation content of construction enterprises will bring a different stock market reaction. Zhao et al. (2021) studied the influence of donation behavior on the stock market performance of enterprises in the context of the COVID-19. The results showed that different donation behaviors of enterprises could bring different market reactions. Enterprises that donated materials related to their primary business had the best stock market performance, and those that donated money had a general market performance. Still, those that did not make donations were penalized by the market. Similarly, Islam et al. (2013) also pointed out that monetary donations are not equally attractive to disaster victims, while shortages of goods are welcomed. Based on this, this paper divides construction firms that make engineering donations and property donations according to the different contents. And the following assumptions are made.

*H1*: Different donation content will bring different stock market reactions; that is, the stock market reaction of construction enterprises with engineering donations is better than that of construction enterprises with property donations.

2. The different natures of corporate equity will lead to different stock market reactions.

Scholars Hoi, Zhang, and Zhuhave showed that the role of charitable donations in promoting corporate performance is more robust in non-state-owned enterprises than in state-owned enterprises (Zhang et al., 2016; Hoi et al., 2019; Zhu and Zhang, 2021). The main reason is that the public expects state-owned enterprises to fulfill their social responsibility more than non-state-owned enterprises. Because state-owned enterprises are founded by the state, belong to public assets, and have the communal nature of assuming more social responsibility and improving social well-being, the social public assigns higher expectations. Based on this, the following assumptions can be made.

*H2*: The differences in the nature of the firm’s equity will bring about differences in stock market response. Thus, the excess returns of non-state-owned construction firms that make donations will be higher than that of state construction firms.

3. Social concern (investor attention) is a mediating variable between corporate donation and stock market performance.

Proactive and timely disclosure of corporate social responsibility fulfillment can generate media coverage, leading to social attention and thus increasing stock returns, which is more evident in sudden crisis events. Fulfilling corporate social responsibility positively affects the number of media reports, which also significantly suppresses the risk of stock collapse (Wu et al., 2021). Some researchers pointed out that investor sentiment and confidence are positively related to stock market response, and high sentiment can effectively improve stock market performance (Irresberger et al., 2015; Kim and Ryu, 2020; Zhai et al., 2021). Information asymmetry exists between enterprises and investors in the market (Peress, 2014; Gao et al., 2020). Still, media coverage can expand the dissemination of information, thus effectively reducing its negative impact, as investors are imperfectly rational with cognitive constraints in behavioral finance theory (Eyster et al., 2019). Investors can only choose to trust the stocks they know better with limited attention, so donation enterprises are more likely to achieve higher stock returns. In early 2020, when the COVID-19 broke out, the rapid response of relevant companies triggered widespread investor attention due to the shortage of epidemic prevention materials. Major media compete in reporting on companies that actively fulfill their social responsibilities, thus increasing social attention and investor attention, affecting stock market performance. Therefore, the following assumptions are made.

*H3a*: The social attention (investor attention) of construction enterprises with different donations will be different; that is, the social attention (investor attention) of construction enterprises with engineering donations is higher than that of construction enterprises with property donations.

*H3b*: Differences in the nature of an enterprise’s equity can lead to differences in investors’ expectations, thus giving non-state-owned enterprises higher social attention (investor attention) than state-owned enterprises.

**Figure 1:**
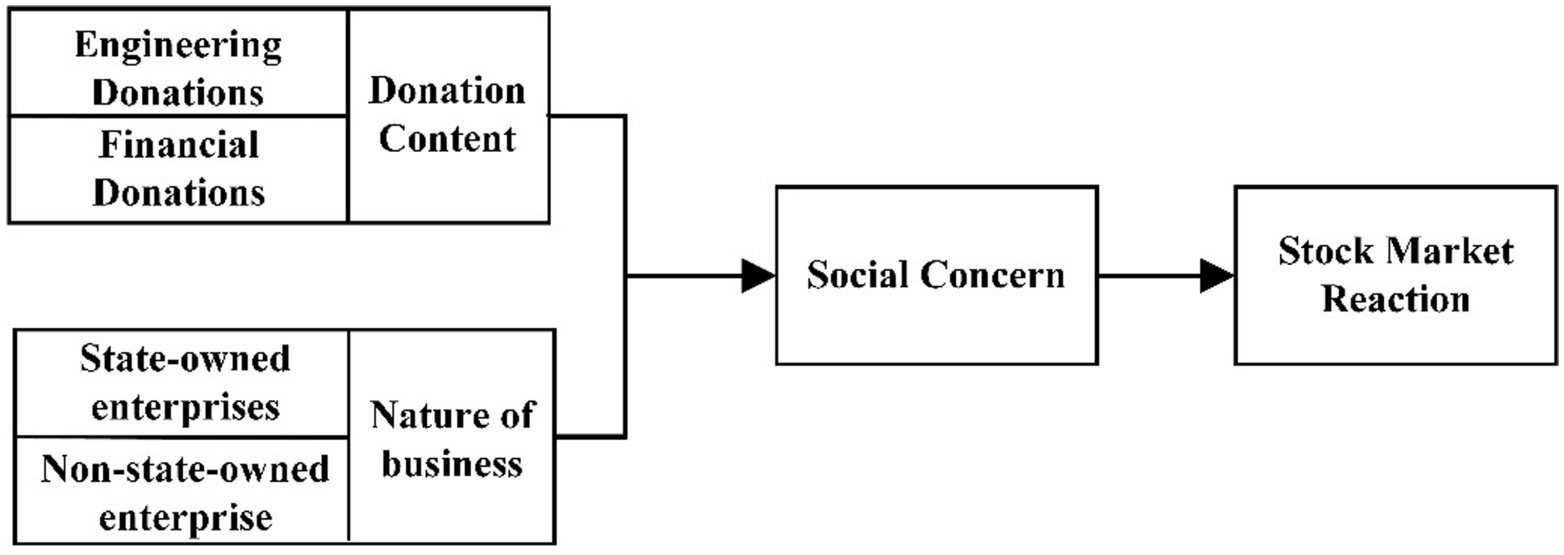
Mechanism of action between emergency donations and stock market returns.

This paper studies the effect of the donation contents and the nature of the equity of the construction companies involved in the donations on the stock excess returns, the path of action of which is a social concern. Therefore, the theoretical framework of this study is shown in Figure 1. Among them, engineering donations refer to the construction enterprises that directly participate in the construction of Vulcan Mountain and Thunder Mountain hospitals, that is, construction enterprises that participate in engineering. Property donation refers to construction companies donating currency or preventing and controlling materials.

## 3. Study design

### 3.1. Research method

This paper uses the event study method and difference-in-difference method for analysis. The event study method is to study the changes in sample stock returns before and after the event and then explain the impact of specific events on sample stock price changes and yields. It is necessary to define the event estimation period and window period to reflect the impact of events on stock price changes during the study period. The stock price of a period before the event can capture the reaction of the capital market on the eve of the event, and the stock price of a period after the event can reflect the attitude of the capital market to such events. The estimation period is defined by using the data for the estimation period to estimate the cumulative rate of return after the event, assuming that no such event occurs. From the actual cumulative rate of return after the event minus the expected cumulative rate of return, the cumulative excess rate of return brought about by the event (Rong et al., 2021). Since the epidemic itself will impact stock market returns, the difference-in-difference method is used to effectively separate the impact of the crisis on stock market returns and more accurately identify the impact of donation content on stock market returns (Tingting et al., 2022).

### 3.2. Data sources

Corporate social responsibility will be announced on the company’s website in time, and as a major social public health event, major news websites will publish donations. This article manually collects donations of A-share listed construction companies during the event window period through listed company announcements, Sina Finance and Baidu News. Due to the small sample size of construction enterprises, according to the 2012 industry classification criteria and certain geographical restrictions, construction material supply, machinery supply, line installation, information technology installation, and other related supply chains were selected.

In this paper, 141 Chinese construction companies (including construction suppliers and machinery manufacturers, etc.) participated in the epidemic donation (excluding ST, PT, and ST* listed companies), of which 41 construction companies made engineering donations. Since the search volume of “pneumonia” in the Baidu Search Index suddenly surged on January 18, 2020, the expert group also conducted the guidance on the scene of COVID-19 on January 18, 2020. Thus this paper selects the week of January 18, 2020 (the third week of 2020) as the event week, and the estimated window period is 120 weeks to 20 weeks before the event week, namely [−120, −20], and the event window is 10 weeks before to last 10 weeks, namely [−10, 10] (He et al., 2020; Weibing and Wingwen, 2021).

Data from November 18, 2019, to April 3, 2020 (the 47th week of 2019 to the 14th week of 2020, with week 5 of 2020 missing due to the Chinese New Year stock market shutdown) were retrieved through the CSMAR (China Stock Market & Accounting Research Database), which contains weekly individual stock return rate, weekly individual stock trading volume, weekly market return rate, and the cumulative death cases per week in each province.

In addition, this paper manually collects information about the donations of the above construction-related companies during the event window, using the week in which the reports or company announcements about the donations could be searched earliest as the donation week. This paper also crawls the relevant construction companies through Python on the Baidu Index website to search the index during the event window.

### 3.3. Variable settings

#### 3.3.1. Explained variables

In this paper, the Cumulative Abnormal Return (CAR) indicator is selected to measure the change in stock returns. The estimation window and the event window are established to calculate the firm’s cumulative abnormal return (CAR) using the event study method (Ma et al., 2021), as shown in the following equation.


(1) $$CAR_{\mathrm{it}}=\sum_{t_{1}}^{t}AR_{it},t\in \left[t_{1},t_{2}\right]$$

(2) $$AR_{\mathrm{it}}=R_{it}-E\left(R_{it}|X_{t}\right)$$

(3) $$E\left(R_{\mathrm{it}}|X_{t}\right)=\overset{\wedge }{\alpha }+\overset{\wedge }{\beta }R_{mt}$$

In the above equation, [ $t_{1}$ , $t_{2}$ ] denotes the event window period. *t* is the time within the event window period, and when *t* = 0, it represents the event week. $AR_{it}$ denotes the abnormal return of stock *i* in week *t*; $R_{\mathrm{it}}$ denotes the actual return of stock *i* in week *t*; $E\left(R_{it}|X_{t}\right)$ denotes the expected estimated return of stock *i* in week *t*, and $R_{mt}$ denotes the weekly market return of each sub-market in week *t* (considering cash dividends reinvested).

#### 3.3.2. Explanatory variables

This paper defines each corporate donation week as “Donation_Date” and introduces the dummy variable “Donation_Code,” set to 1 after the donation week and 0 before the donation week during the event window. Define the “Donation_Content” of the participating construction company as a dummy variable, which takes the value of 1 if it is a construction company making an engineering donation and 0 otherwise, and define the “Ownership” of the participating construction company as a dummy variable, which takes the value of 1 if it is a non-state-owned enterprise, and 0 if it is a state-owned enterprise.

The content of the donation (“Donation_Content”) is interacted with “Donation_Code” to obtain “Content_Code” as the first core explanatory variable in this paper. The nature of the equity of the participating construction companies (“Ownership”) interacted with “Donation_Code” to obtain “Ownership_Code” as the second core explanatory variable in this paper.

In studying the mediating role, the social concern is defined as the “Index,” expressed as the Baidu Search Index of construction companies plus one, and then taken as a logarithm. In conducting the regression, “Content_Code” interacted with “Index” to obtain “Content_Index” as the third core explanatory variable of this paper; “Ownership_Code” interacted with “Index” to obtain “Ownership_Index” as the fourth core explanatory variable of this paper.

#### 3.3.3. Control variables

In addition to the above four core explanatory variables, the cumulative abnormal return of stocks is also influenced by other factors, so the control variables are set as follows: weekly individual stock trading volume (Trading_Volume), expressed as the weekly number of traded shares of individual stocks plus one and taking the logarithm; weekly market return (Mreturn), expressed as the weekly market return of each sub-market (considering cash dividends reinvested); Weekly Cumulative Deaths (C_Dead), expressed as cumulative weekly deaths plus one and taking the logarithm. Since the community’s perception of the epidemic is vague during the outbreak’s peak at the beginning, the fatal cases will bring a more intuitive impact and cause panic among investors. The names and definitions of the above variables are detailed in Table 1.

**Table 1:** Variable names and their definitions.

Variable name	Variable definition
CAR	Cumulative Abnormal Return
Donation_Code	Dummy variable, take 1 after the construction company donation week and 0 before the donation week
Donation_Content	Dummy variable, take 1 for construction companies making engineering donations; otherwise, take 0
Content_Code	Dummy variable, interaction item between Donation_Code and Donation_Content
Ownership	Dummy variables, non-state-owned enterprises take 1, state-owned enterprises take 0
Ownership_Code	Dummy variables, Date_Code, and Ownership interaction items
Index	Social attention, Baidu search index of construction companies during the event window plus 1 and take the logarithm
Content_Index	Interaction items of Content_Code and Index
Ownership_Index	Interaction of Ownership_Code with Index
Trading_Volume	Weekly number of individual shares traded, the number of individual shares traded per week plus 1, and take the logarithm
Mreturn	Weekly market return, weekly market return by sub-market (considering reinvestment of cash dividends)
C_Dead	Cumulative weekly deaths, add 1 to cumulative weekly deaths and take the logarithm

### 3.4. Model design

This paper adopts a combination of the event study method and the difference-in-differences (DID) method. The event study method calculates each stock’s cumulative abnormal return (CAR) during the event window. And secondly, the following model is constructed based on the DID model and the proposed hypotheses H1, H2, and H3.

Model I.


(4) $$CAR_{\mathrm{it}}=\alpha_{1}X+\sum_{j=2}^{4}\alpha_{j}Y_{it}+\sum Date+\sum Enterprise+\varepsilon_{it}$$

Equation (4) $CAR_{\mathrm{it}}$ represents the cumulative abnormal return of stock i from week $t_{1}$ to week t. X represents the first and second core explanatory variables: Content_Code and Ownership_Code, which are used to test the effect of differences in the donation content and the nature of the equity of the participating construction companies on the excess return of the stock.

Model II.


(5) $$CAR_{\mathrm{it}}=\delta_{1}Z+\sum_{j=2}^{4}\delta_{j}Y_{it}+\gamma V+\sum Date+\sum Enterprise+\varepsilon_{it}$$

In Equation (5), Z represents this paper’s third and fourth core explanatory variables: Content_Index and Ownership_Index, which test the mediating effect of social attention, and V represents social attention (Index).

The above two models $Y_{\mathrm{it}}$ represent the same control variables, Trading_Volume, Mreturn, and C_Dead, $\varepsilon_{\mathrm{it}}$ representing the residual term and ΣDate and ΣEnterprise are the time-fixed effects and firm-fixed effects, respectively.

The coefficients of $\alpha_{1}$ and $\delta_{1}$ in the above equation are the two most essential coefficients of the two models. If they are positive, then their corresponding core explanatory variables are positively correlated with CAR; the hypothesis is valid; otherwise, the hypothesis is invalid.

## 4. Empirical analysis

### 4.1. Descriptive statistics

According to the donation content, the two categories are divided into those who make engineering donations and those who make financial donations. The descriptive statistics are presented in Table 2, in which construction companies who make engineering donations account for 29.08%, and the cumulative abnormal return, the number of weekly individual stock transactions, and the social concern are higher; the cumulative weekly death cases in the provinces where construction companies are located cases significantly influenced their willingness to participate in donations; the more cumulative death cases, the higher the willingness to donate.

**Table 2:** Descriptive statistics of donation contents.

Variable name	Engineering donations			Donation of property		
	Sample size	Average value	Standard deviation	Sample size	Average value	Standard deviation
CAR	820	0.022	0.139	2000	0.021	0.146
Donation_Content	820	1	0	2000	0	0
Trading_Volume	820	7.886	0.519	2000	7.607	0.586
Mreturn	820	0.001	0.032	2000	0.001	0.032
C Dead	820	0.779	1.041	2000	0.597	0.876
Ownership	820	0.512	0.500	2000	0.661	0.490
Index	820	8.306	0.935	2000	7.757	1.511

### 4.2. Regression analysis

#### 4.2.1. Regression model

According to Equation (4), the DID estimation results are shown in Table 3. *X* denotes the core explanatory variables, and the first column is Content_Code to verify the different effects of different donation contents by construction firms on their stock markets; the second column is Ownership_Code to test the effects of differences in the nature of equity of construction firms involved in donations on their stock markets. Relevant control variables are also included, with time-fixed effects and firm-fixed effects.

**Table 3:** Regression results of donation content and nature of business on CAR.

Variable name	CAR	
	Content_Code	Ownership_Code
*X*	0.0378*** (0.00617)	0.0151*** (0.00524)
Mreturn	0.135 (0.184)	0.144 (0.185)
Trading_Volume	0.192*** (0.00689)	0.193*** (0.00692)
C_Dead	−0.0128*** (0.00309)	−0.00925*** (0.00306)
Constant	−1.439*** (0.0526)	−1.448*** (0.0528)
Date	Yes	Yes
Enterprise	Yes	Yes
Observations	2,820	2,820
*R*-squared	0.718	0.715

***, **, * indicate significant at the 1%, 5%, and 10% levels, respectively.

It can be seen that the regression coefficients of the two core explanatory variables in Model 1 are both significantly positive at the 1% level, indicating that construction firms that make engineering donations have substantially higher abnormal returns than that make financial donations; non-state-owned firms that make donations are more recognized by investors than state-owned firms and receive higher abnormal returns. Meanwhile, Trading_Volume is significantly and positively correlated with CAR, indicating that the more weekly individual stocks are traded, the higher their cumulative abnormal returns; C_Dead is significantly and negatively correlated with CAR, and the more cumulative deaths, the lower the abnormal returns, indicating that the severity of the epidemic will bring negative emotions and lower confidence to investors.

The regression analysis of model 2 was conducted according to Equation (5), and its regression results are shown in Table 4 to test the mediating role of social concern. Concerning the Weibing and Wingwen (2021), Z denotes the core explanatory variables, the first column is Content_Index, and the second column is Ownership_Index. Relevant control variables are also included, with time-fixed effects and firm-fixed effects. It can be seen that the regression coefficients of both core explanatory variables in Model 2 are significantly positive at the 1% level, indicating that both construction firms that made engineering donations and non-state-owned construction firms that participated in donations receive more social attention and thus better stock market responses.

**Table 4:** Path test for the role of social concern.

Variable name	CAR	
	Content_Index	Ownership_Index
*Z*	0.00651*** (0.000737)	0.00209*** (0.000672)
Index	0.0244*** (0.00285)	0.0241*** (0.00288)
Mreturn	0.0441 (0.210)	0.0535 (0.213)
Trading_Volume	0.178*** (0.00759)	0.179*** (0.00770)
C_Dead	−0.0116*** (0.00326)	−0.00584* (0.00324)
Constant	−1.541*** (0.0574)	−1.547*** (0.0581)
Date	Yes	Yes
Enterprise	Yes	Yes
Observations	2,820	2,820
*R*-squared	0.719	0.712

By analyzing the regression results above, H1, H2, and H3 were significant at the 1% level and passed the test.

#### 4.2.2. Robustness test

##### 4.2.2.1. Additional control variables

Additional control variables are chosen to ensure the robustness of the results to prevent endogenous problems. When there are fewer control variables, the significance may be due to endogenous problems. After increasing the control variables, it remains significant, which can better reflect the validity and reliability of the conclusion. By reviewing the relevant literature, it is found that firm size (Size, total assets taken as logarithm), gearing (Lev), and operating capacity (Turnover, the ratio of operating income to total assets) also affect the cumulative abnormal return of firms. Social concern is also introduced; individual and time-fixed effects are also performed. The regression results are shown in Table 5. The CAR coefficient is found to remain significant, indicating the robustness of the results.

**Table 5:** Adding control variables.

Variable name	CAR	
	Content_Code	Ownership_Code
*X*	0.0512*** (0.00664)	0.0172*** (0.00597)
Index	0.0330*** (0.00318)	0.0330*** (0.00318)
mreturn	0.109 (0.228)	0.109 (0.228)
trading_volume	0.0871*** (0.00595)	0.0871*** (0.00595)
C_Dead	−0.00414 (0.00349)	−0.00414 (0.00349)
Size	0.00144 (0.0131)	0.00144 (0.0131)
Lev	0.355*** (0.101)	0.355*** (0.101)
Turnover	0.161*** (0.0313)	0.161*** (0.0313)
Constant	−2.157*** (0.311)	−1.241*** (0.320)
Observations	2,820	2,820
*R*-squared	0.733	0.682

##### 4.2.2.2. Counterfactual ideas

In order to ensure the reliability of the behavioral condition setting of epidemic donation, this paper conducts a “virtual behavior” setting at different periods to test its effectiveness and reliability. The occurrence time of the event is set virtually to see whether there is a significant difference between the control and experimental groups. If there is no significant difference, it passes the robustness test. This paper assumes that the event occurrence week is 20 weeks before the actual occurrence week; namely, the 36th week of 2019 is the virtual occurrence week, and its first 6 weeks and last 5 weeks are the sample interval. The regression analysis is conducted for the model I. The results are shown in Table 6. It can be seen that none of the CAR coefficients are significant, indicating that before the “epidemic donation” behavior, there was no significant difference in the cumulative excess return rate of construction enterprises with different donation contents and different equity natures. It can draw that the reason for the later difference is due to the “epidemic donation” behavior, indicating that the results reflect the robustness and reliability of the hypothesis and data analysis.

**Table 6:** Robustness tests of counterfactual ideas.

Variable name	CAR	
	Content_Code	Ownership_Code
*X*	0.00474 (0.00377)	−0.000404 (0.00342)
Mreturn	−0.203 (0.169)	−0.206 (0.169)
Trading_Volume	0.0489*** (0.00235)	0.0490*** (0.00235)
Constant	−0.855*** (0.0401)	−0.856*** (0.0401)
Date	Yes	Yes
Enterprise	Yes	Yes
Observations	1,630	1,630
*R*-squared	0.838	0.838

Through the above tests, it can be seen that the hypothesis-testing conclusion of this paper is robust and reliable.

### 4.3. Study results

This paper takes the donation of construction enterprises and the construction of Vulcan Mountain and Thunder Mountain in the COVID-19 epidemic as research samples for empirical analysis. This paper collects the donation information of 141 listed construction enterprises that donated during the epidemic and put forward hypotheses based on the previous literature review and theoretical development. Using the event study method and the difference-in-difference method for regression analysis, it is concluded that H1, H2, and H3 all pass the test and, at the same time, pass the robustness test, indicating that the research results are robust and reliable. This paper finds that non-state-owned enterprises have higher social attention than state-owned enterprises in emergency donation and have better stock market performance, which is consistent with the conclusion of Zhu and Zhang (2021). This paper also draws a new conclusion, in the construction of emergency projects, construction enterprises with engineering participation will obtain more social concern and, therefore, better stock market response. This is consistent with the conclusion of Zhao et al. (2021) that the stock market performance of enterprises that donate materials related to their main business is the best, and the market performance of enterprises that donate money is average. The positive mediating role of social attention between emergency donations and stock market performance has also been tested, consistent with that of Gao et al. (2020).

## 5. Discussion and recommendations

Construction of emergency engineering requires construction contractors and cooperation between the government and enterprises (Stringfellow, 2014; Pribadi et al., 2018; Sospeter et al., 2020). Similarly, another scholar pointed out that building multi-agent coordination and process-balanced emergency management mechanism is necessary to realize the modernization of the emergency management system and governance capacity (Haibo, 2020). The construction of emergency hospitals during the COVID-19 epidemic has developed toward the trend of “government-enterprise” cooperation and the participation of multiple subjects, such as the public, achieving a win-win situation for society and enterprises. The organizational model of engineering construction has also played a demonstration role in constructing future emergency projects. The framework for the emergency engineering construction mode is proposed with the research results.

Firstly, research shows that construction companies that engaged in engineering participation have better stock market performance because the construction of Vulcan Mountain and Thunder Mountain is the key to epidemic prevention of the epidemic, which has received extensive attention from the public, and in turn, triggers the attention of investors so that participating enterprises obtain higher excess returns. Therefore, construction enterprises can improve their professionalism in emergency projects and use their advantages to participate in engineering. Due to the urgency of emergency projects, the traditional bidding mode does not meet the practical needs, so groups of professional and socially responsible construction enterprises are needed to conduct professional engineering participation. From hypothesis H1, it can be concluded that construction enterprises with engineering participation can improve their stock market returns and competitiveness and, thus, increase their enthusiasm to involve in emergency engineering donations in the future. Therefore, the participation of construction enterprises in emergency projects through donation mode can significantly improve the speed of emergency projects, alleviate the government’s pressure, and achieve a win-win situation between society and enterprises.

**Figure 2:**
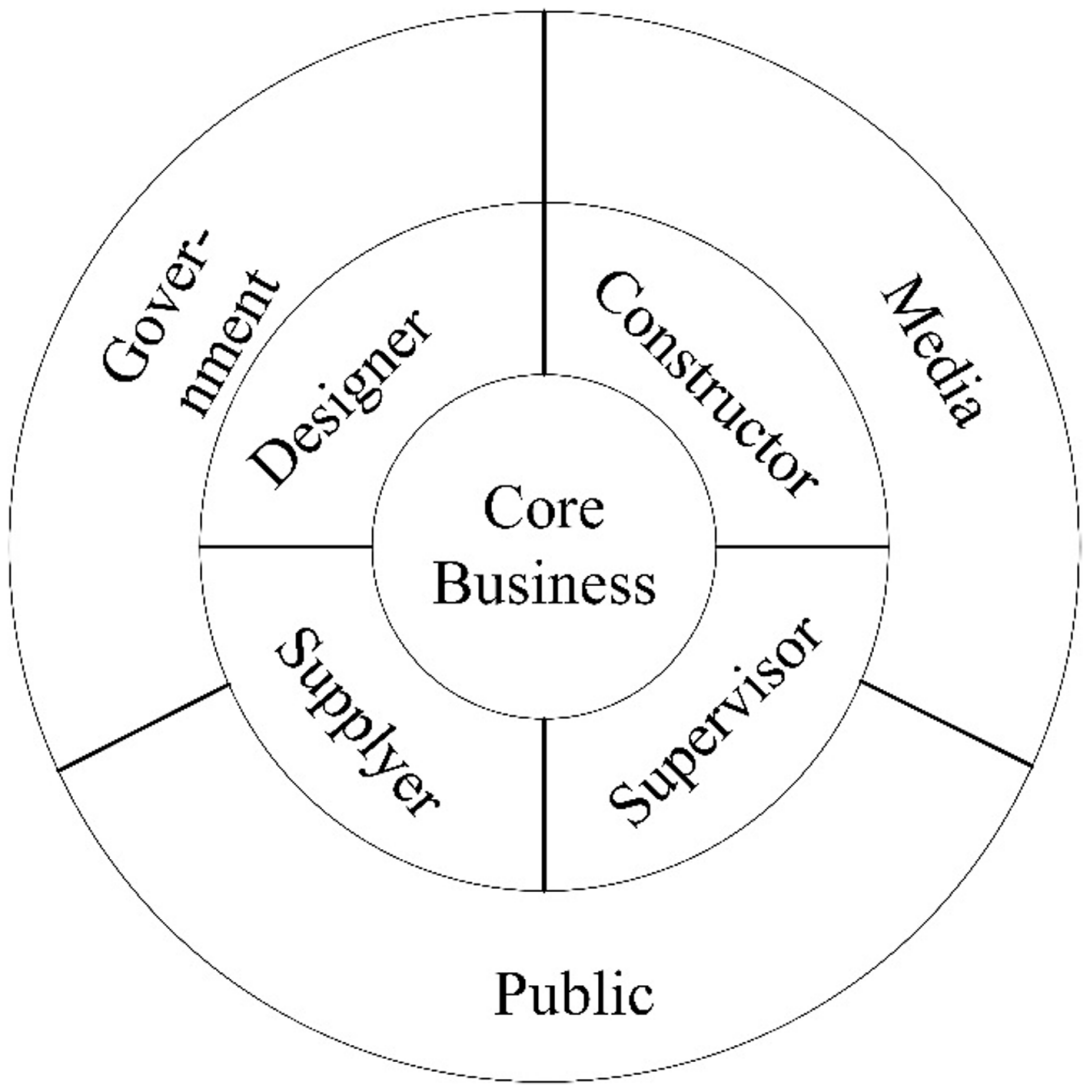
“Engineering Community” system.

Secondly, the excess returns of non-state-owned construction enterprises participating in emergency donations are significantly higher than those of state-owned construction enterprises. State-owned enterprises are public assets and have the communal nature of assuming more social responsibility and improving social welfare, so emergency donations by non-state-owned enterprises receive more attention and recognition from investors, thereby improving stock returns. Therefore, in the construction of emergency projects, state-owned construction enterprises should undertake more engineering donations. Large state-owned enterprises should exert their inherent cohesion and organizational power to lead other state-owned enterprises and encourage non-state-owned enterprises to participate in the construction. In the COVID-19 epidemic, Vulcan Mountain and Thunder Mountain were led by the government, and China State Construction Engineering Group Co., Ltd. (CSCEC) was appointed as the general contractor to be responsible for the whole process, forming an “engineering community” (Nanjun et al., 2021). Under the organization of CSCEC, its subsidiaries, local enterprises, and other large state-owned enterprises also completed the preliminary preparation projects such as communication, power transmission, and drawing design for the first time. The core enterprise, CSCEC, organized and coordinated other construction enterprises to carry out construction activities centered on the needs of construction projects, optimize resource allocation, coordinate construction organizations, and build a multi-functional and hierarchical emergency construction system (Yanhu and Xingxu, 2021). In addition to the government-led mobilization and the positive response of enterprises, the media’s follow-up and the public’s support are also essential components of the “engineering community” (Nanjun et al., 2021). The framework of the construction model of emergency projects is shown in Figure 2, dominated by the core enterprise. The government, media, and the public support the collaborative cooperation among the organization design side, the supply side, the construction site, and the supervision side, providing ideas for the organization model of emergency projects management.

Thirdly, social attention positively mediates between the emergency donations of construction enterprises and the improvement of stock returns. As a result, construction enterprises should take the initiative to increase information disclosure while participating in professional emergency donations to correct information asymmetry in the market, improving social attention, their reputation capital, and stock market performance, thus achieving the effect of strategic donations. In the face of sudden disasters for the construction of emergency projects, enterprises should emphasize their professionalism rather than a specific donation amount when making relevant donation disclosures because social attention focuses on the donation of services rather than the property itself. State-owned enterprises should pay more attention to their organizational capabilities and the timeliness of their participation in donations to show social responsibility and transmit more accurate information to the public.

**Figure 3:**
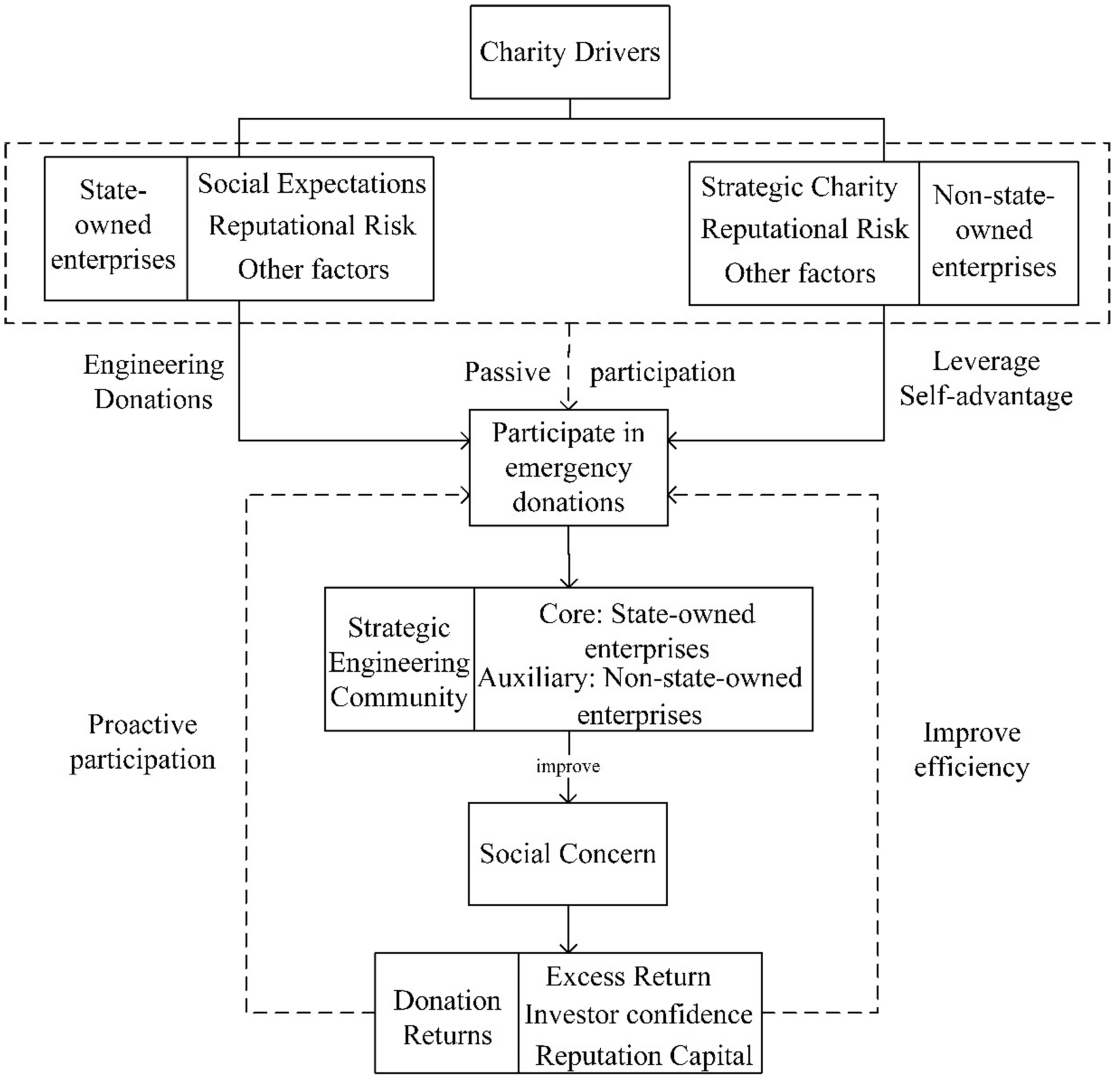
Logic diagram of construction companies’ participation in emergency donations.

Fourthly, the governance logic of the emergency engineering construction model driven by donation incentives is shown in Figure 3. Due to the suddenness and urgency of emergency projects, construction enterprises with relevant experience and technology are required to participate in the construction, So for enterprises that have participated in similar projects in the past, they can directly participate in the construction by “invitation,” which can improve the speed of response and the efficiency and quality of emergency projects construction. In addition, after the emergency donation, the feedback and rewards from society can bring stock market returns and improve intangible capital, such as the enterprises’ reputation and competitiveness (Stringfellow, 2014). In turn, it can maintain the enthusiasm of construction enterprises to participate in emergency donations and motivate other construction enterprises to actively fulfill their social responsibilities, forming a “virtuous circle” and truly achieving a normal win-win development of society and enterprises.

## 6. Conclusion

This paper focuses on the economic effect of construction enterprises after emergency donation and the organizational model of emergency engineering management. Through empirical analysis and hypothetical inference methods, this paper explores the framework ideas and basis of the organizational model of emergency engineering construction to stimulate construction enterprises to participate in engineering donation and let them take the initiative to participate in the construction of emergency engineering, thus improving the construction quality and efficiency of emergency engineering.

This paper believes that professional donation is the key to the efficient construction of emergency projects. Secondly, the future development trend of emergency projects is to have a balanced process and multi-agent collaboration, so this paper innovatively puts forward the concept of “engineering community” strategic development. The core of the “engineering community” is multi-collaboration. The emergency donation will bring stock returns, social concerns, investor trust, and reputation capital to enterprises. And emergency projects are time-consuming and arduous. A strategic “engineering community” can effectively solve these difficulties through experience in similar projects and trust in multiple cooperation. It can improve the construction efficiency of emergency projects and maintain the enthusiasm of construction enterprises to participate in emergency donations to motivate other construction enterprises to participate in emergency charity, form a virtuous circle, and build a diversified emergency construction management system.

### 6.1. Implications

The first contribution of this paper is that it makes up for the research gap of the heterogeneity of emergency donation content and the economic effect after donation, especially in emergency construction. This paper proposes a new donation model – “engineering participation” donation. The second contribution is to enrich the development of emergency management theory further. In this paper, emergency engineering construction and emergency donation are coupled theoretically. In this paper, the construction enterprises involved in engineering will build an “engineering community” and put forward the logical model of its strategic development. The possibility is discussed, and a new model for construction management of future emergency works is proposed. At the same time, at a practical level, when the normalized “engineering community” is applied in future emergency projects, the construction of emergency projects will be guaranteed with high efficiency and high quality; and participating enterprises will also receive social incentives, realizing a win-win situation between society and enterprises.

### 6.2. Limitation and futuristic approach

However, this paper also has certain limitations. This paper only selects one disaster for empirical research, and future research can conduct comparative research under the background of multiple disasters. At the same time, under the new model, the interactive interface relationship between enterprises, government, and other social organizations to achieve a win-win path between society and enterprises is an issue that needs further research in the future.

## Data availability statement

The original contributions presented in the study are included in the article/Supplementary material, further inquiries can be directed to the corresponding author.

## Author contributions

HL conceptualized and wrote the original draft. XZ supervised. UK participated in reviewing and editing. FR did the formal analysis. All authors contributed to the article and approved the submitted version.

## Conflict of interest

The authors declare that the research was conducted in the absence of any commercial or financial relationships that could be construed as a potential conflict of interest.

## Publisher’s note

All claims expressed in this article are solely those of the authors and do not necessarily represent those of their affiliated organizations, or those of the publisher, the editors and the reviewers. Any product that may be evaluated in this article, or claim that may be made by its manufacturer, is not guaranteed or endorsed by the publisher.
