# Supplementary material for: Emergency engineering reconstruction mode based on the perspective of professional donations
Source: Front Psychol. 2023 Jan 17;14:971552. doi: 10.3389/fpsyg.2023.971552 (PMC9887034; doi:10.3389/fpsyg.2023.971552)
Supplement: Supplementary file 1 [file Data_Sheet_2.docx]

Supplementary Material

**Figures**


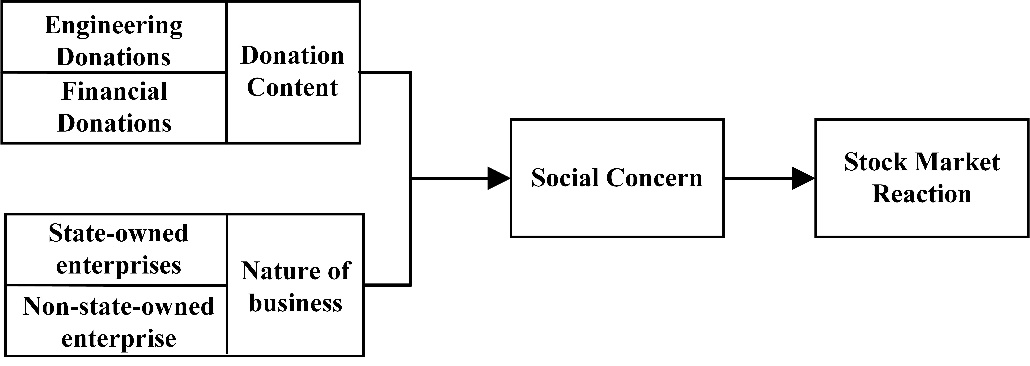


Figure 1 Mechanism of action between emergency donations and stock market returns


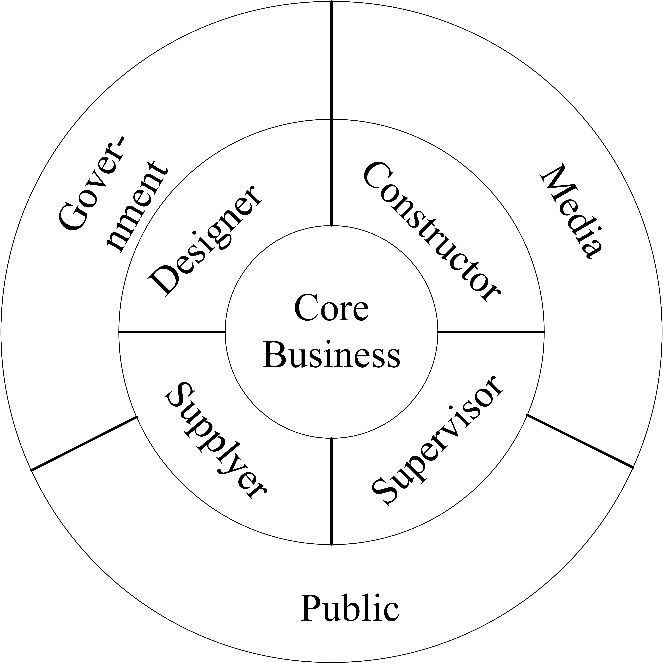


Figure 2 "Engineering Community" system


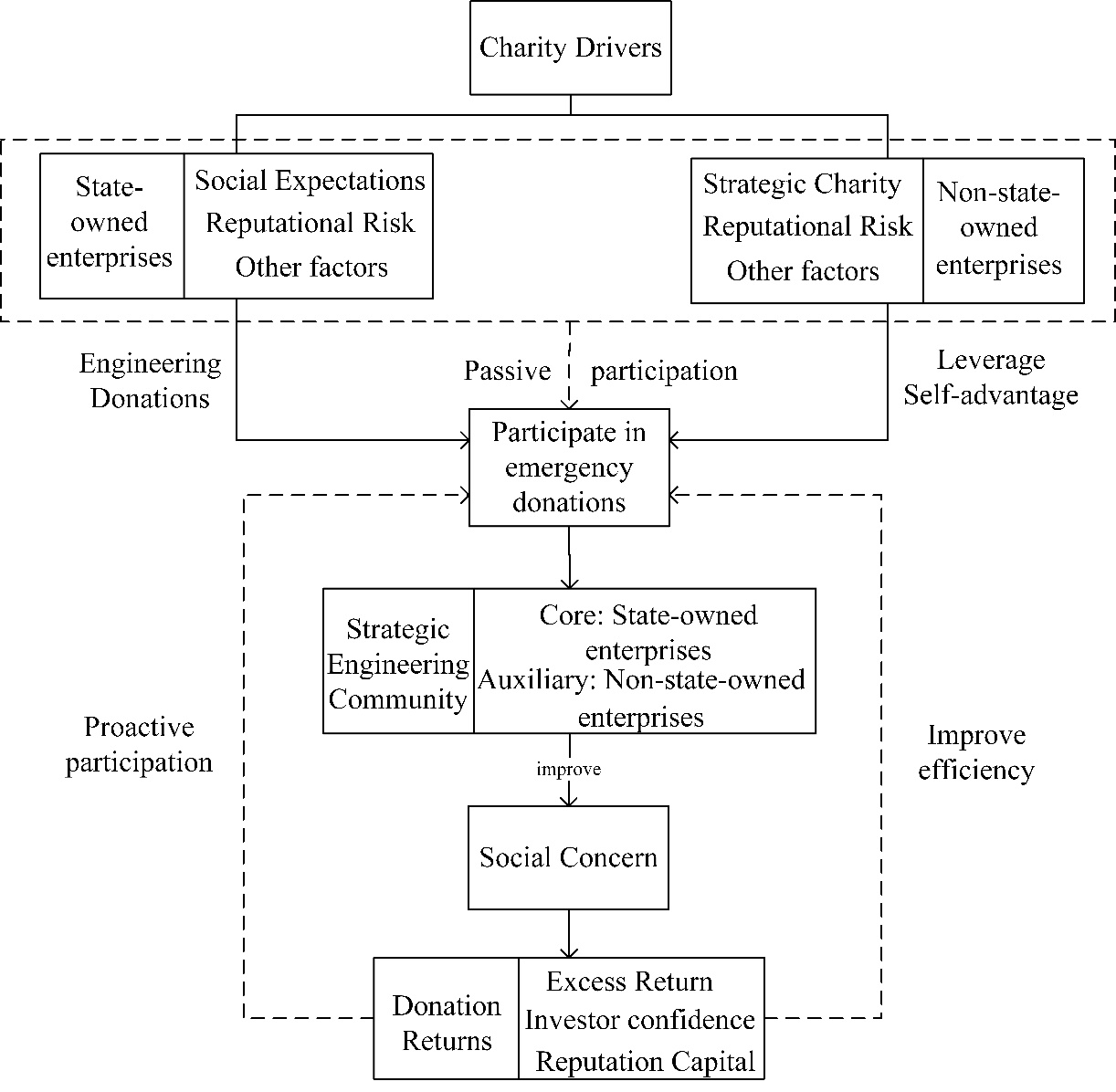


Figure 3 Logic diagram of construction companies' participation in emergency donations

**Tables**

Table 1 Variable names and their definitions

| Variable Name | Variable Definition |
| --- | --- |
| CAR | Cumulative Abnormal Return |
| Donation_Code | Dummy variable, take 1 after the construction company donation week and 0 before the donation week |
| Donation_Content | Dummy variable, take 1 for construction companies making engineering donations; otherwise, take 0 |
| Content_Code | Dummy variable, interaction item between Donation_Code and Donation_Content |
| Ownership | Dummy variables, non-state-owned enterprises take 1, state-owned enterprises take 0 |
| Ownership_Code | Dummy variables, Date_Code, and Ownership interaction items |
| Index | Social attention, Baidu search index of construction companies during the event window plus 1 and take the logarithm |
| Content_Index | Interaction items of Content_Code and Index |
| Ownership_Index | Interaction of Ownership_Code with Index |
| Trading_Volume | Weekly number of individual shares traded, the number of individual shares traded per week plus 1, and take the logarithm |
| Mreturn | Weekly market return, weekly market return by sub-market (considering reinvestment of cash dividends) |
| C_Dead | Cumulative weekly deaths, add 1 to cumulative weekly deaths and take the logarithm |

Table 2 Descriptive statistics of donation contents

| Variable Name | Engineering Donations | | | Donation of property | | |
| --- | --- | --- | --- | --- | --- | --- |
|  | Sample size | Average value | Standard deviation | Sample size | Average value | Standard deviation |
| CAR | 820 | 0.022 | 0.139 | 2000 | 0.021 | 0.146 |
| Donation_Content | 820 | 1 | 0 | 2000 | 0 | 0 |
| Trading_Volume | 820 | 7.886 | 0.519 | 2000 | 7.607 | 0.586 |
| Mreturn | 820 | 0.001 | 0.032 | 2000 | 0.001 | 0.032 |
| C Dead | 820 | 0.779 | 1.041 | 2000 | 0.597 | 0.876 |
| Ownership | 820 | 0.512 | 0.500 | 2000 | 0.661 | 0.490 |
| Index | 820 | 8.306 | 0.935 | 2000 | 7.757 | 1.511 |

Table 3 Regression results of donation content and nature of business on CAR

| Variable Name | CAR | |
| --- | --- | --- |
|  | Content_Code | Ownership_Code |
| X | 0.0378***  (0.00617) | 0.0151***  (0.00524) |
| Mreturn | 0.135  (0.184) | 0.144  (0.185) |
| Trading_Volume | 0.192***  (0.00689) | 0.193***  (0.00692) |
| C_Dead | -0.0128***  (0.00309) | -0.00925***  (0.00306) |
| Constant | -1.439***  (0.0526) | -1.448***  (0.0528) |
| Date | Yes | Yes |
| Enterprise | Yes | Yes |
| Observations | 2820 | 2820 |
| R-squared | 0.718 | 0.715 |

***, ** , * indicate significant at the 1%, 5%, and 10% levels, respectively

Table 4 Path test for the role of social concern

| Variable Name | CAR | |
| --- | --- | --- |
|  | Content_Index | Ownership_Index |
| Z | 0.00651***  (0.000737) | 0.00209***  (0.000672) |
| Index | 0.0244***  (0.00285) | 0.0241***  (0.00288) |
| Mreturn | 0.0441  (0.210) | 0.0535  (0.213) |
| Trading_Volume | 0.178***  (0.00759) | 0.179***  (0.00770) |
| C_Dead | -0.0116***  (0.00326) | -0.00584*  (0.00324) |
| Constant | -1.541***  (0.0574) | -1.547***  (0.0581) |
| Date | Yes | Yes |
| Enterprise | Yes | Yes |
| Observations | 2,820 | 2,820 |
| R-squared | 0.719 | 0.712 |

Table 5 Adding control variables

| Variable Name | CAR | |
| --- | --- | --- |
|  | Content_Code | Ownership_Code |
| X | 0.0512***  (0.00664) | 0.0172***  (0.00597) |
| Index | 0.0330***  (0.00318) | 0.0330***  (0.00318) |
| mreturn | 0.109  (0.228) | 0.109  (0.228) |
| trading_volume | 0.0871***  (0.00595) | 0.0871***  (0.00595) |
| C_Dead | -0.00414  (0.00349) | -0.00414  (0.00349) |
| Size | 0.00144  (0.0131) | 0.00144  (0.0131) |
| Lev | 0.355***  (0.101) | 0.355***  (0.101) |
| Turnover | 0.161***  (0.0313) | 0.161***  (0.0313) |
| Constant | -2.157***  (0.311) | -1.241***  (0.320) |
| Observations | 2820 | 2,820 |
| R-squared | 0.733 | 0.682 |

Table 6 Robustness tests of counterfactual ideas

| Variable Name | CAR | |
| --- | --- | --- |
|  | Content_Code | Ownership_Code |
| X | 0.00474  (0.00377) | -0.000404  (0.00342) |
| Mreturn | -0.203  (0.169) | -0.206  (0.169) |
| Trading_Volume | 0.0489***  (0.00235) | 0.0490***  (0.00235) |
| Constant | -0.855***  (0.0401) | -0.856***  (0.0401) |
| Date | Yes | Yes |
| Enterprise | Yes | Yes |
| Observations | 1630 | 1630 |
| R-squared | 0.838 | 0.838 |
